# Estimates of Species Richness and Composition Depend on Detection Method in Assemblages of Terrestrial Mammals

**DOI:** 10.3390/ani11010186

**Published:** 2021-01-14

**Authors:** Bruno D. Suárez-Tangil, Alejandro Rodríguez

**Affiliations:** Department of Conservation Biology, Estación Biológica de Doñana—CSIC, Américo Vespucio 26, 41092 Sevilla, Spain; bdstangil@gmail.com

**Keywords:** consistency, efficiency, large-scale, mammal communities, rapid surveys, sampling effort

## Abstract

**Simple Summary:**

Transformation of forest into farmland and many other drivers of global change have the potential for quickly reducing or altering the set of mammal species inhabiting a particular place. To document this process, lists of species updated at regular intervals should be compared. Several detection techniques are available to make lists of medium to large mammal species during field surveys. We explore whether four field methods differ in their efficiency to describe a complete list of species and in their capacity to produce the same list, provided that species composition has not changed. Using track surveys, we detected all species present in a region encompassing three Mediterranean landscapes and obtained the most accurate description of the number of species in 24 specific localities within the region. The sampling effort needed for track surveys was also relatively low. Had we chosen camera traps, scent stations, or scat surveys as the only survey method during the same period, we would have obtained incomplete species lists. We show that the common practice of using a single detection method without previous evaluation may produce unreliable species inventories, hampering a correct assessment of the impact of human activity on wild mammals.

**Abstract:**

Detecting rapid changes in mammal composition at large spatial scales requires efficient detection methods. Many studies estimate species composition with a single survey method without asking whether that particular method optimises detection for all occurring species and yields reliable community-level indices. We explore the implications of between-method differences in efficiency, consistency, and sampling effort for the basic characterisation of assemblages of medium to large mammals in a region with three contrasted Mediterranean landscapes. We assessed differences between camera traps, scent stations, scat surveys, and track surveys. Using track surveys, we detected all species present in the regional pool (13) and obtained the most accurate description of local species richness and composition with the lowest sampling effort (16 sampling units and 2 survey sessions at most). Had we chosen camera traps, scent stations, or scat surveys as the only survey method, we would have underestimated species richness (9, 11, and 12 species, respectively) and misrepresented species composition in varying degrees. Preliminary studies of method performance inform whether single or multiple survey methods are needed and eventually which single method might be most appropriate. Without such a formal assessment current practices may produce unreliable and incomplete species inventories, ultimately leading to incorrect conclusions about the impact of human activity on mammal communities.

## 1. Introduction

The scale at which human activity impacts on biodiversity, manifested through processes such as quick colonisation and transformation of remote areas, atmospheric warming, or the spread of invasive species, is quickly shifting from local to regional or global [1,2]. The impacts of human activity produce changes in the composition of mammal assemblages over large regions, for example through the extinction of sensitive species or the invasion of exotic species, unbalancing ecological interactions, homogenising species composition, or prompting the loss of ecosystem services [3,4]. Medium to large wild terrestrial mammals, hereafter referred to as mammals, are involved in important ecosystem functions as predators [5], herbivores [6], and seed dispersers [7]. As the extent and intensity of human activity increase at an exponential rate, regional changes in the composition of mammal assemblages are expected to accelerate. Documenting community dynamics is needed not only to investigate causal mechanisms [8] but also to guide conservation measures that could alleviate regional impacts [9], e.g., regulating the extraction of wildlife or designing nature reserve networks. Detecting rapid changes in mammal composition requires frequent monitoring [10], considerable replication [11], and efficient detection methods capable of providing reliable estimates during short operation periods [12].

The trade-off between extent and sampling intensity determines, in cases when the size of the area to be surveyed is large, the need for efficient and quick detection of every occurring species. Characterising mammal communities over large, heterogeneous regions implies intensive spatial replication. This is because landscape or habitat stratification should be considered to cover the range of environmental conditions. As species differ in their degree of habitat specialisation, a large enough number of localities should be sampled in each stratum to increase the chances of detecting every species. However, the number of observers and/or available equipment is typically limited by economic and labour costs [13], precluding all replicates from being sampled at the same time. Rather, batches of sites have to be sampled sequentially [14,15], which implies that sampling periods are expected to be as short as possible in order to minimise the whole span of the survey and the associated temporal variation in environmental conditions. Therefore, in the context of rapid, large-scale surveys, quick species detection becomes an essential attribute of method performance.

All field methods show some degree of specificity in species detection; that is, each method tends to detect some species better or quicker than others [16,17]. Further, as different survey methods may also show different efficiency (their ability to detect as many species as possible during a given operation period) and consistency (their ability to keep a high efficiency over sampling replicates), multiple, complementary survey methods are often used to increase the chances that every mammal species in the assemblage is detected [18,19]. Species-specificity of detection methods introduces a new trade-off, because the use of several methods involves not only a higher cost but also longer times of deployment, handling, and checking, thus reducing the agility needed in rapid surveys. Consequently, the number of detection methods should be minimised in order to speed up surveys and to cover the study region during short time windows, making rapid, large-scale mammal surveys feasible [14]. It follows that the use of a single or a few methods whose efficiency and consistency have not been previously tested may produce a misleading description of the mammal assemblage.

The composition of mammal communities is simply expressed as a list of species. At the regional, continental or global scales, lists are built using a variety of sources such as opportunistic records [20], citizen science [21], museum collections [22], bibliographical material [23] or systematic field surveys [24] for a number of purposes, for example preparing mammal atlases. Given the logistical difficulties of performing frequent surveys across large areas, communities are usually characterised by combining distribution maps for single species, and each map is typically the product of cumulating heterogeneous records [25]. The gathering of records over enough time provides a reasonable approximation to regional species pools [26], but species lists from distribution maps are of little use to track quick changes in species composition [27]. At the other end of the spectrum, field surveys accurately reporting changes in the composition of mammal communities often provide results only in the short term and at a local scale [28,29]. In between, efforts to monitor mammal communities on the intermediate spatiotemporal scales meaningful for detecting the effects of the drivers of global change are uncommon. Moreover, with a few exceptions (e.g., [30,31]), method efficiency and consistency are seldom assessed, and the effect of choosing a given field method to detect mammals on community indices has received little attention. Without such critical evaluation, surveys may yield incorrect community indices and a poor evaluation of the effects of human activity on mammal communities.

In this paper, we explore the implications of between-method differences in efficiency and consistency for the characterisation of mammal communities, and provide a novel framework for evaluating method performance, optimising the estimation of simple community-level indices for rapid regional mammal inventories. Firstly, we evaluate the efficiency of four widely used field detection methods, namely camera traps, scent stations, scat surveys, and track surveys [32,33,34]. We compare how much species composition and species richness estimated with each detection method approaches corresponding values derived from a multi-method approach, i.e., the joint application of the four methods. We also calibrate observed and estimated species richness against the known composition of the regional species pool. Secondly, we compare method reliability by measuring whether simple community indices are consistent over multiple survey attempts. Thirdly, we quantify the survey effort needed to detect the entire assemblage using each detection method. Finally, we assess whether any detection method stands out more than the others based on these criteria and can, therefore, be proposed for rapid species inventories of Mediterranean mammal communities across large areas.

## 2. Materials and Methods

### 2.1. Study Area

**Figure 1:**
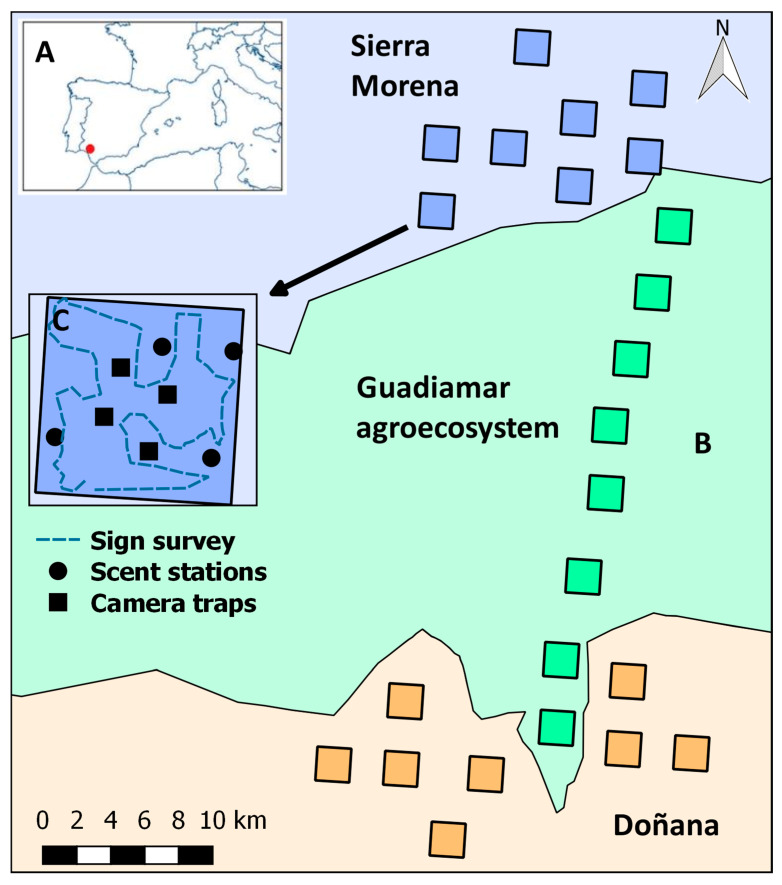
Study area. (**A**) Location of the study area in the lower Guadiamar basin, SW Spain. (**B**) Sketch of the study area showing the distribution of 4 km^2^ sampling units (*n* = 24) among three distinct landscapes (Sierra Morena, Guadiamar agroecosystem, and Doñana). (**C**) Distribution of sites with camera traps and scent stations, and an example of a sign survey conducted within one sampling unit.

Our study was carried out in a 900 km^2^ region in the lower basin of the Guadiamar river, south-western Spain (37°21′ N–6°13′ W; Figure 1A). The climate is Mediterranean with Atlantic type influences. This region encompasses three landscapes differing in land use and structure: Sierra Morena, the Guadiamar agroecosystem, and the periphery of the Doñana nature reserve (Figure 1B). The upstream landscape of Sierra Morena consists of a mosaic of Mediterranean forest, tree plantations, scrubland, and “dehesa” (pasturelands with open oak woodland; [35]). Major land uses include forestry, livestock husbandry, and big game hunting. The agroecosystem occupies a flat open valley between Sierra Morena and Doñana, where the dominant land use is agricultural (cereal crops, olive groves, and fruit tree plantations), and natural vegetation is restricted to thin strips of riparian forests, hedgerows and small, degraded, and scattered woodlots [36]. The riparian vegetation of the Guadiamar River is protected. The downstream landscape of Doñana is a flat area occupied by a diverse mosaic of scrubland, pine forests, pastureland, and crops.

### 2.2. Sampling Design and Methods

We focused on mammals (body mass: 0.8–160 kg) known to occur in the study area [37]. Lagomorphs include the European rabbit (*Oryctolagus cuniculus*) and the Iberian hare (*Lepus granatensis*). Wild boar (*Sus scrofa*) and red deer (*Cervus elaphus*) are the only representatives of the order Cetartiodactyla. Nine species of the order Carnivora occur in the region, namely red fox (*Vulpes vulpes*), common genet (*Genetta genetta*), Egyptian mongoose (*Herpestes ichneumon*), Eurasian badger (*Meles meles*), stone marten (*Martes foina*), Eurasian otter (*Lutra lutra*), European polecat (*Mustela putorius*), Iberian lynx (*Lynx pardinus*) and wildcat (*Felis silvestris*).

We employed four commonly used survey methods for inventorying mammals: camera traps, scent stations, track surveys, and scat surveys. In each landscape, we established eight 4 km^2^ square sampling units separated at least 2 km from each other (Figure 1B). We considered these units as independent spatial replicates. In each sampling unit, we identified favourable places (trails, clearings, or crossroads) for mammal detection with passive methods and selected eight sites or stations separated as much as possible from one another (Figure 1C). We placed stations spaced a mean distance of 537 m (range 293–2485 m). Spread of stations helped to sample the environmental heterogeneity present within sampling units. Four scent stations and four camera traps were randomly allocated to the eight stations. We sampled mammals twice a year for two consecutive years (2000–2001), in late spring and late autumn, i.e., four sessions or surveys. Monitoring of each sampling unit lasted ten days. As we could not afford the eight sampling units simultaneously we deployed scent stations and camera traps in part of the units with a delay of a few days in order to complete fieldwork for each landscape within two weeks. Time restrictions allowed us to cover the whole area during a period short enough to minimise changes in environmental conditions, home range occupancy, species abundance, and the probability of detecting transient individuals.

Mechanically triggered analogue cameras (Canon Prima BF-9s; Canon Inc., Ōta City, Japan) were placed at the height of 20 cm inside an open wooden case and hidden in vegetation whenever possible. Camera traps were activated by pressure plates (33.0 × 25.0 × 0.3 cm) detecting mammals heavier than 0.8 kg and this threshold defines the set of species potentially detected in this study. Pressure plates were protected from soil and moisture by inserting them into plastic bags, and were shallowly buried in the ground in front of cameras at a distance of 1.5–3.0 m. Camera traps were operated for ten consecutive days in each session (total effort of 24 sampling units × 4 cameras/unit × 10 days/session × 4 sessions = 3840 camera-days). Cameras were checked for proper function and film replacement on days 1, 2, 6, and 10. Mammal species were easily identified in pictures. Two pictures of the same species should be shot with a difference of >5 min to be considered separate records. Scent stations consisted of circular layers of sifted sand or earth 0.9 m in diameter and 2–4 cm thick [38]. Scent stations were operated for two consecutive nights at the beginning of each session (total effort of 768 scent station-days). In the morning, we checked the status, identified species from tracks, and smoothed the surface to allow the identification of new prints. All stations were lured with olfactory attractants and half of the stations in each sampling unit were also lured with visual and/or auditory stimuli. We used two different olfactory lures (Pocatello Supply Depot USDA, Pocatello, ID, USA): plaster discs saturated with fatty acid scent and calcium sulphate prisms soaked with catnip oil. The visual lure was a piece of silver tinsel and handcrafted wooden wind bells were used as the auditory lure. Further details about the design and the use of the attractants can be found elsewhere [39]. Within the limits of the sampling units, an observer searched for mammal scats and tracks along paths over 90 min (Figure 1C), inspecting places where sign finding was most likely, taking into account the habitat preferences of all 13 species (e.g., mammal trails, muddy areas, unpaved roads). For species using latrines, or producing pellet piles, aggregated faecal remains were considered a single record. We counted tracks as separate records only if they belonged to different trails. Attributes of scats and tracks markedly differ among target species [40], which eased identification. Fieldwork was performed only by experienced observers in order to minimise the probability of sign misidentification. Any doubtful assignment of signs to species was discarded. Scat surveys and track surveys were conducted once per sampling unit and session (total effort of 24 sampling × 4 sessions = 96 sign-surveys).

Different methods were operated during different times because either increasing operation time did not substantially increase species detection (scent stations, sign surveys; [41,42]) or the maximum operation time was imposed by the duration of each session (camera traps). As some species are detected with a particular method better than others, multiple methods are often used jointly to estimate species richness [12,15], that is, the survey method consists in a combination of several detection methods. We also considered that a species was detected by a fifth method called ‘multi-method’ when the species was detected by one or more of the four survey methods described above.

### 2.3. Analyses

We recorded the occurrence of mammal species by each survey method in each session and sampling unit. As the regional species pool was well known in the study area [37], we employed observed species richness as a response variable. A separate value of observed species richness was recorded per each detection method in each sampling unit and session. Given the high spatiotemporal replication of our field surveys, we assumed that the best estimate of species richness and species composition in each sampling unit was derived from the multi-method survey. Henceforth we considered these estimates as the true values against which comparing the efficiency of each method. Nevertheless, we also estimated species richness to simulate uncertainty about the actual composition of the regional species pool. We calculated iChao2 index [43] to estimate species richness employing “ChaoSpecies” function from SpadeR package [44].

To analyse pairwise differences in observed species richness between methods, we carried out a Kruskal–Wallis rank sum test applying the Bonferroni correction. We calculated multiple pairwise comparisons between methods by performing a Tukey and Kramer test. To analyse the effect of survey method performance on species richness, we used generalised linear models with a Poisson distribution. Spatio-temporal factors such as environmental conditions and season determine the distribution, abundance and activity levels of mammals and, consequently, these factors influence which species are detected as well as estimates of species richness and composition. Therefore, we controlled for the potential effects of spatio-temporal factors on species richness, including the effect of landscape type and sampling season into the models. We fitted all possible models combining three explanatory variables: landscape type, sampling season, and survey method. To generate the models, we used the “dredge” function from MuMIn package [45]. Model selection was based on the corrected AIC values (AIC_c_). We selected models with the lowest AIC_c_ value, and models with ΔAIC_c_ < 2 were considered competitive [46]. When two or more models were competitive we built a weighted average model using the “model.avg” function from MuMIn package. We quantified differences in species richness between methods by calculating the odds ratio, which indicates how efficiently the species richness was described by each survey method in comparison to a reference method. To analyse the consistency of methods, we followed the same procedure and carried out a set of generalised linear models for each session.

We analysed the spatial and temporal replication effort needed to inventory member species in the mammal assemblage with each survey method. We built species accumulation curves by adding sampling units in random order up to 24 to examine between-method differences in the spatial replication effort required to detect a given number of species. Similarly, we constructed species accumulation curves by adding sessions in random order up to 4 to analyse between-method differences in the temporal replication effort needed to detect a given number of species. To find the mean species richness and its standard error from random permutations of the data, we used 9999 random replicates for both spatial and temporal accumulation curves. Species accumulation curves were generated using the “specaccum” function from Vegan package [47].

We compared species composition derived from each detection method and the best estimate of the actual composition of each local assemblage, i.e., the species composition resulting from the multi-method survey. To assess consistency, we compared differences in species composition derived from the same method between different sessions. We calculated the Jaccard incidence-based similarity index using the “beta.pair” function from betapart package [48]. All statistical analyses were carried out using R software [49].

## 3. Results

Each detection method yielded a different estimate of species composition of the mammal assemblage as well as different estimates of species richness (Table S1). For all methods except scat surveys, species richness estimated with iChao2 index greatly overestimated the known richness of the regional species pool. Depending on the survey method, the estimated number of species exceeded actual regional richness by 14–58% (mean 32%) for spatial replicates and 5–58% (mean 30%) for temporal replicates (Table S1). Given this consistent bias in richness estimates, in further analyses we only considered cumulative species counts from raw observed richness.

**Figure 2:**
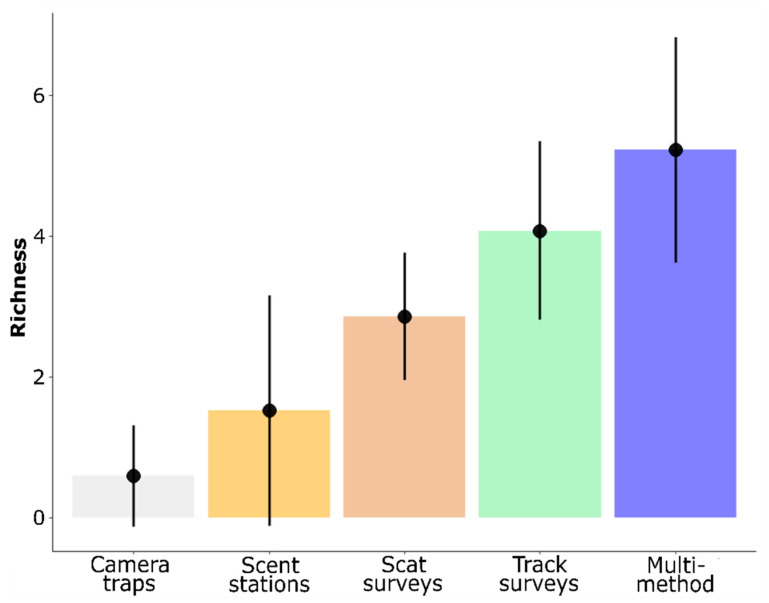
Mean (±SD) species richness of Iberian mammals observed from four survey methods and the multi-method survey in the lower Guadiamar basin (*n* = 96 per method, i.e., 24 sampling units × 4 sessions).

Species occurrence over the 24 sampling units, as described by the multi-method survey, matched the composition of the regional species pool (13 species; Table 1). Considering specific sampling methods, we only detected all 13 species using track surveys. Mean species richness recorded with the multi-method survey was 8.8, 3.4, 1.8 and 1.3 times higher than mean species richness recorded with camera traps, scent stations, scat surveys, and track surveys, respectively (Figure 2). We found significant differences between survey methods in the number of species detected (Kruskal–Wallis χ^2^ = 327.2, df = 4, *p* < 0.001; Figure 2). Differences between all pairs of methods were significant (post-hoc tests in Table S2). Between-method differences in species richness were consistent in all four sessions of the study period (Figure S1): spring of year 1 (Kruskal–Wallis χ^2^ = 87.86, df = 4, *p* ≤ 0.001), autumn of year 1 (Kruskal–Wallis χ^2^ = 77.91, df = 4, *p* ≤ 0.001), spring of year 2 (Kruskal–Wallis χ^2^ = 82.35, df = 4, *p* ≤ 0.001) and autumn of year 2 (Kruskal–Wallis χ^2^ = 82.46, df = 4, *p* ≤ 0.001). These differences were significant at the Bonferroni-corrected significance level of α = 0.0125. Species richness observed with camera traps was consistently and significantly lower than richness observed with scat surveys, track surveys, or the multi-method survey, whereas species richness observed with scent stations was lower than richness observed with track surveys or the multi-method survey (post-hoc test in Table S3).

Two generalised linear models of species richness were selected (Table 2). In the averaged model, the effect of survey method was significant. Odds ratio values indicated that species richness derived from track surveys was significantly higher than richness resulting from camera traps, scent stations or scat surveys (Figure S2). After accounting for the effect of landscape type and sampling season (Table 2), species richness observed with scent stations, scat surveys, track surveys, and multi-method surveys were 2.6, 4.8, 6.9, and 8.8 times higher than species richness observed with camera traps (Figure S2). The effect of the survey method on species richness was consistent in all four replicates throughout the study period (Figure S3; Table S4).

**Figure 3:**
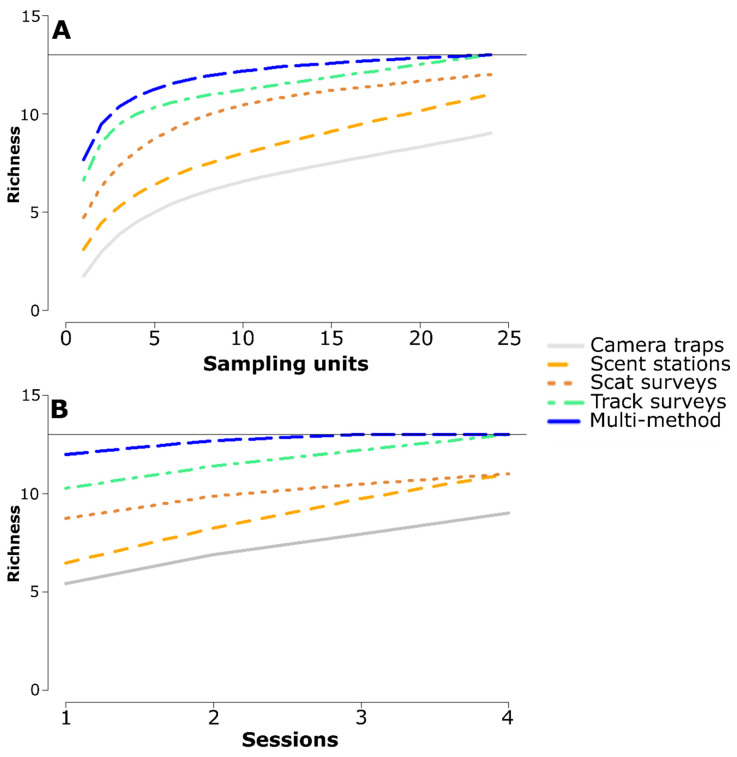
Estimated accumulation curves of species richness built with data from different survey methods as a function of effort in terms of (**A**) spatial replicates surveyed across the study area, and (**B**) temporal replicates six months apart. The horizontal line indicates the actual richness of the regional species pool.

Species accumulation curves showed that track surveys detected more species than camera traps, scent stations, and scat surveys for any given effort in terms of the number of sampling units surveyed (Figure 3A). The maximum species richness recorded by camera traps, scent stations, and scat surveys across 24 sampling units was obtained with track surveys after applying a sampling effort of just 3, 9, and 16 units, respectively. The maximum species richness recorded by the multi-method survey was obtained with track surveys after sampling 24 units (Figure 3A). Determining species richness with track surveys also required a lower number of sessions than with any other detection method (Figure 3B). Maximum species richness recorded by camera traps, scent stations, and scat surveys after 4 sessions was attained by track surveys in 1, 2, and 2 sessions, respectively. The maximum species richness recorded by the multi-method survey was obtained with track surveys after four sessions (Figure 3B).

Mean (±SD) dissimilarity in species composition between the 24 local mammal assemblages described by each method and the assemblage described by the whole set of survey methods was 0.51 (±0.19) for camera traps, 0.43 (±0.17) for scent stations, 0.22 (±0.04) for scat surveys, and 0.09 (±0.07) for track surveys. Further, the consistency of estimates of community composition during different survey occasions varied across detection methods. Specifically, mean (±SD) dissimilarity in species composition between sessions for each method was 0.43 (±0.13) for camera traps, 0.44 (±0.06) for scent stations, 0.25 (±0.14) for scat surveys and 0.19 (±0.09) for track surveys.

## 4. Discussion

Sampling efficiency is an essential component in the design of strategies for monitoring the dynamics of communities [50]. Our framework for comparing sampling efficiency considers three aspects that are often overlooked during the assessment of different mammal detection methods. The first aspect is inspecting not only whether methods are capable of detecting as many species as possible but also how precise they are, their ability to yield consistent results throughout temporal replicates [51]. As precise methods would require a lower number of survey repeats to describe mammal assemblages at a given point in time, consistency is an important attribute of method performance considering the time constraints of rapid, large-scale community monitoring. Secondly, it is desirable to control for spatio-temporal fluctuations (represented by season and landscape type in our study) to isolate the influence of sampling method on observed species richness [52]. Spatio-temporal factors capture major variation in environmental conditions, influence species probability of detection and, consequently, may lead to inaccurate estimates of species richness and composition [53]. The uncertainty introduced by spatio-temporal factors is expected to be stronger when large, heterogeneous landscapes are sampled during long periods. Thirdly, similarity in species composition tends to be neglected in the assessment of method efficiency, which often focuses on species richness (e.g., [54,55]). However, species richness alone prevents knowing whether different methods detect exactly the same set of species or, alternatively, equally rich but quite different subsets of species in the assemblage [56]. Hence, it is advisable to explicitly consider species composition when comparing the efficiency of sampling methods.

Species lists resulting from species distribution maps or biodiversity databases are sometimes the only available source for estimating simple community indices over large regions. This kind of information may constitute a reasonable approximation to the regional species pool [26], but it is unsuitable for evaluating rapid shifts in mammal communities [57,58]. This is because data are accumulated over very long periods (typically several decades), obtained from quite heterogeneous sources and, sometimes, after applying an uneven sampling effort across space. Given the considerable attention paid to the mammals of the study region, we are confident that actual richness is known (13 species), in agreement with atlas cumulative data [37]. Species richness calculated with the non-parametric index iChao2 clearly overestimated true richness in contrast with recent analyses indicating that iChao2 tends to underestimate richness both in virtual and real communities with a larger number of species (20–125 species; [59]). Although Tingley et al. [59] concluded that iChao2 was an accurate estimator, they also showed that accuracy notably decreases when mean species probability of occupancy is low (0.1) and highly variable (SD = 1.0), regardless of variation in the average species probability of detection. However, we found considerable overestimation of species richness even with high probabilities of occupancy (mean ± SD = 0.77 ± 0.37; *n* = 13 species) which are closer to scenarios where iChao2 performed better [59]. Observed species richness is usually thought to underestimate species richness, as increasing sampling effort increases the number of species observed until reaching an asymptote [60]. Our results show that, although estimating richness has been recommended in most situations [59,61], in some cases the observed species richness may approach true richness more closely than estimated richness, even using a moderate number of temporal replicates (four sessions in our study).

The observed richness recorded using the multi-method survey best resembled true richness of the regional species pool. The joint use of all four methods also yielded the maximum richness in each locality. These results support the hypothesis that single methods seldom provide a complete description of mammal assemblages, which is partly due to method’s species-specificity [62,63] and partly the consequence of a biased detection towards the most conspicuous or abundant species [53,55]. Nevertheless, there are still numerous examples of large-scale mammal surveys that use a single detection method for characterising mammal communities without explicitly testing its efficiency and consistency [64,65,66], thus overlooking the risk of underestimating community indices.

We found substantial differences in species richness and composition obtained with alternative survey methods. Although previous studies have noticed such differences in community indices (e.g., [31,67,68]), the magnitude of differences in method efficiency has rarely been quantified and, more importantly, the potential implications of imprecise estimates of richness and composition are generally disregarded. To our knowledge, only three studies have formally compared and discussed the effect of survey methods on simple community indices and its implications for mammal inventorying. Cromsigt et al. [33] suggested that scat surveys better represented herbivore diversity in Hluhluwe-iMfolozi Park (South Africa), recommending scat surveys for future monitoring designs instead of more conventional approaches such as sightings. Swan et al. [69] found that only the combination of multiple detection methods provided a consistent description of the mammal assemblage occurring in Otway Ranges (southeastern Australia). Finally, Fragoso et al. [70] also pointed out that sightings underdetected terrestrial mammals in southern Guyana in comparison to sign surveys, which might have direct consequences on community-based wildlife management. Our results strongly suggest that reliable data describing communities should be collected with methods whose efficiency and consistency have been previously evaluated. Ignoring the relative efficiency and consistency of alternative methods may lead to the underestimation of species richness and composition [71,72] and, ultimately, to wrong conclusions when the impact of human activity on mammal assemblages is under examination.

Whereas mammal communities could be better characterised by the joint deployment of multiple detection methods, this approach is also expensive and time-consuming, limiting the size of the area that can be simultaneously covered. As sampling time should be minimised in rapid, large-scale mammal surveys, it is convenient to identify and use a single efficient method wherever possible [14,32]. Probably not every method has the potential for a high efficiency given the constraints of rapid surveys. For example, camera traps could better approach true species composition only after extended operation periods [73] whereas there is indication that accuracy in species composition estimates might not substantially improve by prolonging the operation period of scent stations [34]. We found that track surveys were the only method that adequately described true mammal richness and composition after applying a moderate sampling effort (24 spatial replicates repeated four times). Therefore, in agricultural Mediterranean landscapes similar to those we sampled, track surveys may be the most efficient and consistent method for a rapid and straightforward description of mammal communities.

## 5. Conclusions

Our results emphasise the need for an informed choice between single and multiple survey methods before any rapid characterisation of mammal assemblages. Combining the results from all four methods maximised efficiency and consistency of community-level parameters. However, the use of a single detection method favours quick surveys. Had we chosen camera traps, scent stations, or scat surveys as the only survey method, we would have underestimated species richness and misrepresented species composition. Our assessment identified track surveys as the most suitable sampling method for monitoring quick changes in the mammal communities of Mediterranean ecosystems. Ignoring how inconsistent or inefficient the chosen sampling methods are may produce unreliable and incomplete species inventories which may ultimately lead to incorrect conclusions about the impacts of human activities on mammal communities.

## Figures and Tables

**Table 1:** The number of sessions (N) in which mammal species were detected (+) by each survey method and by the joint use of all detection methods. The species list contains all wild terrestrial mammals heavier than 0.8 kg known to occur in the study area [37]. Sessions—S1: year 1, spring; S2: year 1, autumn; S3: year 2, spring; S4: year 2, autumn.

	Track Surveys					Scat Surveys					Scent Stations					Camera Traps					Multi-Method				
Species	S1	S2	S3	S4	N	S1	S2	S3	S4	N	S1	S2	S3	S4	N	S1	S2	S3	S4	N	S1	S2	S3	S4	N
European rabbit	+	+	+	+	4	+	+	+	+	4	+	+	+	+	4	+	+	+	+	4	+	+	+	+	4
Iberian hare	+	+	+	+	4	+	+	+	+	4	+	+	+	+	4	+	+	+		3	+	+	+	+	4
Wild boar	+	+	+	+	4	+	+		+	3				+	1					0	+	+	+	+	4
Red deer	+	+	+	+	4	+	+	+	+	4	+				1	+				1	+	+	+	+	4
Red fox	+	+	+	+	4	+	+	+	+	4	+	+	+	+	4	+	+	+	+	4	+	+	+	+	4
Common genet	+	+	+	+	4	+	+	+	+	4	+	+		+	3	+	+	+	+	4	+	+	+	+	4
Egyptian mongoose	+	+	+	+	4			+		1		+		+	2	+	+	+		3	+	+	+	+	4
Eurasian badger	+	+	+	+	4	+	+			2	+	+	+	+	4	+				1	+	+	+	+	4
Stone marten			+		1	+	+	+	+	4		+			1		+			1	+	+	+	+	4
Eurasian otter	+	+	+	+	4	+	+	+	+	4					0					0	+	+	+	+	4
European polecat			+		1			+		1		+			1		+			1		+	+		2
Iberian lynx			+		1				+	1					0					0			+	+	2
Wildcat	+			+	2					0	+				1					0	+			+	2

**Table 2:** Effect of survey method on species richness. (a) Competitive generalised linear models (ΔAIC_c_ ≤ 2) ordered by the fit statistic AIC_c_. Models differ in the external factors that are controlled for, i.e., sampling season and landscape. (b) Parameter estimates of the resulting averaged model. The method ‘Camera trap’, the season ‘Autumn’, and the landscape ‘Sierra Morena’ are included in the intercept. AIC_c_: corrected Akaike Information Criterion; w_i_: Akaike weights.

	Model	df	AIC_c_	ΔAIC_c_	w_i_
(a)	Survey method + Landscape + Season	8	1537.9	0.00	0.608
	Survey method + Landscape	7	1538.9	1.02	0.365
(b)		**Effect**	**Coefficient**	**SE**	***p***
		Intercept	−0.437	0.141	0.002
		Scent station	0.941	0.156	<0.001
		Scat survey	1.570	0.146	<0.001
		Track survey	1.926	0.142	<0.001
		Multi-method	2.174	0.140	<0.001
		Guadiamar agroecosystem	−0.217	0.066	0.001
		Doñana	−0.140	0.065	0.031
		Spring	0.095	0.054	0.080

## Data Availability

The data presented in this study are available in Table 1.

## Supplementary Materials

The following are available online at https://www.mdpi.com/2076-2615/11/1/186/s1. Figure S1: Mean (±SD) mammal species richness of local mammal assemblages obtained from four survey methods and the multi-method survey in each session. Figure S2: Odds ratio, as an index of the relative efficiency of survey methods for describing species richness; Figure S3: Consistency across sessions of odds ratios quantifying between-method differences in detection of all species present in each local community as compared to the reference method; Table S1: Observed vs. estimated mammal species richness obtained from each survey method; Table S2: Pair-wise comparisons of the species richness described by different detection methods using Tukey and Kramer test with Tukey distance approximation (α = 0.05); Table S3: Pair-wise differences in species richness during the sampling period; Table S4: Effect of survey method on species richness for each session.

## References

[1] Canale G.R., Peres C.A., Guidorizzi C.E., Gatto C.A.F., Kierulff M.C.M. (2012). Pervasive defaunation of forest remnants in a tropical biodiversity hotspot. PLoS ONE.

[2] Reidsma P., Tekelenburg T., van den Berg M., Alkemade R. (2006). Impacts of land-use change on biodiversity: An assessment of agricultural biodiversity in the European Union. Agric. Ecosyst. Environ..

[3] Nielsen T.F., Sand-Jensen K., Dornelas M., Bruun H.H. (2019). More is less: Net gain in species richness, but biotic homogenization over 140 years. Ecol. Lett..

[4] Cardinale B.J., Duffy J.E., Gonzalez A., Hooper D.U., Perrings C., Venail P., Narwani A., Mace G.M., Tilman D., Wardle D.A. (2012). Biodiversity loss and its impact on humanity. Nature.

[5] Díaz-Ruiz F., Delibes-Mateos M., García-Moreno J.L., López-Martín J.M., Ferreira C., Ferreras P. (2013). Biogeographical patterns in the diet of an opportunistic predator: The red fox *Vulpes vulpes* in the Iberian Peninsula. Mamm. Rev..

[6] Rueda M., Rebollo S., Gálvez-Bravo L., Escudero A. (2008). Habitat use by large and small herbivores in a fluctuating Mediterranean ecosystem: Implications of seasonal changes. J. Arid. Environ..

[7] Fedriani J.M., Delibes M. (2009). Functional diversity in fruit-frugivore interactions: A field experiment with Mediterranean mammals. Ecography.

[8] Rich L.N., Davis C.L., Farris Z.J., Miller D.A.W., Tucker J.M., Hamel S., Farhadinia M.S., Steenweg R., di Bitetti M.S., Thapa K. (2017). Assessing global patterns in mammalian carnivore occupancy and richness by integrating local camera trap surveys. Glob. Ecol. Biogeogr..

[9] Dorji S., Rajaratnam R., Falconi L., Williams S.E., Sinha P., Vernes K. (2018). Identifying conservation priorities for threatened Eastern Himalayan mammals. Conserv. Biol..

[10] Rovero F., Ahumada J. (2017). The Tropical Ecology, Assessment and Monitoring (TEAM) network: An early warning system for tropical rain forests. Sci. Total Environ..

[11] Laméris D.W., Tagg N., Kuenbou J.K., Sterck E.H.M., Willie J. (2020). Drivers affecting mammal community structure and functional diversity under varied conservation efforts in a tropical rainforest in Cameroon. Anim. Conserv..

[12] Rossi R.V., Miranda C.L., Semedo T.B.F. (2016). Rapid assessment of nonvolant mammals in seven sites in the northern State of Pará, Brazil: A forgotten part of the Guiana Region. Mammalia.

[13] Waldron A., Mooers A.O., Miller D.C., Nibbelink N., Redding D., Kuhn T.S., Roberts J.T., Gittleman J.L. (2013). Targeting global conservation funding to limit immediate biodiversity declines. Proc. Natl. Acad. Sci. USA.

[14] Jambari A., Sasidhran S., Halim H.R.A., Mohamed K.A., Ashton-Butt A., Lechner A.M., Azhar B. (2019). Quantifying species richness and composition of elusive rainforest mammals in Taman Negara National Park, Peninsular Malaysia. Glob. Ecol. Conserv..

[15] Nader M.R., El Indary S., Abi Salloum B., AbouDagher M. (2011). Combining non-invasive methods for the rapid assessment of mammalian richness in a transect-quadrat survey scheme—Case study of the Horsh Ehden Nature Reserve, North Lebanon. Zookeys.

[16] Velli E., Bologna M.A., Silvia C., Ragni B., Randi E. (2015). Non-invasive monitoring of the European wildcat (*Felis silvestris silvestris* Schreber, 1777): Comparative analysis of three different monitoring techniques and evaluation of their integration. Eur. J. Wildl. Res..

[17] Long R.A., Donovan T.M., MacKay P., Zielinski W.J., Buzas J.S. (2007). Comparing scat detection dogs, cameras, and hair snares for surveying carnivores. J. Wildl. Manag..

[18] Calaça A., Fachi M., Silva D.A., Oliveira S.R., de Melo F.R. (2019). Mammals recorded in isolated remnants of Atlantic Forest in southern Goiás, Brazil. Biota Neotrop..

[19] Azad M.A.J.B.A.G. (2006). Mammal diversity and conservation in a secondary forest in Peninsular Malaysia. Biodivers. Conserv..

[20] Massimino D., Harris S.J., Gillings S. (2018). Evaluating spatiotemporal trends in terrestrial mammal abundance using data collected during bird surveys. Biol. Conserv..

[21] Danielsen F., Burgess N.D., Balmford A. (2005). Monitoring matters: Examining the potential of locally-based approaches. Biodivers. Conserv..

[22] Gaubert P., Papeş M., Peterson A.T. (2006). Natural history collections and the conservation of poorly known taxa: Ecological niche modeling in central African rainforest genets (*Genetta* spp.). Biol. Conserv..

[23] Linzey D.W. (2016). Mammals of Great Smoky Mountains National Park: 2016 revision. Southeast. Nat..

[24] Lesmeister D.B., Nielsen C.K., Schauber E.M., Hellgren E.C. (2015). Spatial and temporal structure of a mesocarnivore guild in Midwestern North America. Wildl. Monogr..

[25] Chi Y., Wang J., Xi C., Qian T., Sheng C. (2020). Spatial pattern of species richness among terrestrial mammals in China. Diversity.

[26] Souza Y., Gonçalves F., Lautenschlager L., Akkawi P., Mendes C., Carvalho M.M., Bovendorp R.S., Fernandes-Ferreira H., Rosa C., Graipel M.E. (2019). ATLANTIC MAMMALS: A data set of assemblages of medium- and large-sized mammals of the Atlantic Forest of South America. Ecology.

[27] Escribano N., Ariño A.H., Galicia D. (2016). Biodiversity data obsolescence and land uses changes. PeerJ.

[28] Easter T., Bouley P., Carter N. (2020). Intraguild dynamics of understudied carnivores in a human-altered landscape. Ecol. Evol..

[29] Gomes L.d.P., Rocha C.R., Brandão R.A., Marinho-Filho J. (2015). Mammal richness and diversity in Serra do Facão region, Southeastern Goiás state, central Brazil. Biota Neotrop..

[30] Santos F.D.S., Mendes-Oliveira A.C. (2012). Diversidade de mamíferos de médio e grande porte da região do rio Urucu, Amazonas, Brasil. Biota Neotrop..

[31] Munari D.P., Keller C., Venticinque E.M. (2011). An evaluation of field techniques for monitoring terrestrial mammal populations in Amazonia. Mamm. Biol..

[32] Orban B., Kabafouako G., Morley R., Gaugris C.V., Melville H., Gaugris J. (2018). Common mammal species inventory utilizing camera trapping in the forests of Kouilou Département, Republic of Congo. Afr. J. Ecol..

[33] Cromsigt J.P.G.M., van Rensburg S.J., Etienne R.S., Olff H. (2009). Monitoring large herbivore diversity at different scales: Comparing direct and indirect methods. Biodivers. Conserv..

[34] Norris D., Peres C.A., Michalski F., Hinchsliffe K. (2008). Terrestrial mammal responses to edges in Amazonian forest patches: A study based on track stations. Mammalia.

[35] Campos P., Huntsinger L., Pro J.L.O., Starrs P.F., Diaz M., Standiford R.B., Montero G. (2013). Mediterranean Oak Woodland Working Landscapes.

[36] Pereira M., Rodríguez A. (2010). Conservation value of linear woody remnants for two forest carnivores in a Mediterranean agricultural landscape. J. Appl. Ecol..

[37] Palomo L.J., Gisbert J., Blanco J.C. (2007). Atlas y Libro Rojo de Los Mamíferos Terrestres de España.

[38] Linhart S.B., Knowlton F.F. (1975). Determining the relative abundance of coyotes by scent station lines. Wildl. Soc. Bull..

[39] Suárez-Tangil B.D., Rodríguez A. (2017). Detection of Iberian terrestrial mammals employing olfactory, visual and auditory attractants. Eur. J. Wildl. Res..

[40] Navarro B.S. (2012). Huellas y Rastros de los Mamíferos de la Península Ibérica.

[41] Carreras-Duro J., Moleón M., Barea-Azcón J.M., Ballesteros-Duperón E., Virgós E. (2016). Optimization of sampling effort in carnivore surveys based on signs: A regional-scale study in a Mediterranean area. Mamm. Biol..

[42] Travaini A., Laffitte R., Delibes M. (1996). Determining the relative abundance of European red foxes by scent-station methodology. Wildl. Soc. Bull..

[43] Chiu C.-H., Wang Y.-T., Walther B.A., Chao A. (2014). An improved nonparametric lower bound of species richness via a modified good-turing frequency formula. Biometrics.

[44] Chao A., Ma K.H., Hsieh T.C., Chiu C.-H. (2016). SpadeR: Species-Richness Prediction and Diversity Estimation with R. https://CRAN.R-project.org/package=SpadeR.

[45] Barton K. (2018). MuMIn: Multi-Model. Inference. https://CRAN.R-project.org/package=MuMIn.

[46] Burnham K.P., Anderson D.R. (2002). Model Selection and Multimodel Inference. A Practical Information-Theoretic Approach.

[47] Oksanen J., Blanchet F.G., Friendly M., Kindt R., Legendre P., McGlinn D., Minchin P.R., O’Hara R.B., Simpson G.L., Solymos P. (2019). Vegan: Community Ecology Package. https://cran.rproject.org/web/packages/vegan/index.html.

[48] Baselga A., Orme C.D.L. (2012). Betapart: An R package for the study of beta diversity. Methods Ecol. Evol..

[49] R Core Team (2020). R: A Language and Environment for Statistical Computing.

[50] Buckland S.T., Johnston A. (2017). Monitoring the biodiversity of regions: Key principles and possible pitfalls. Biol. Conserv..

[51] Valente A.M., Binantel H., Villanua D., Acevedo P. (2018). Evaluation of methods to monitor wild mammals on Mediterranean farmland. Mamm. Biol..

[52] Suárez-Tangil B.D., Rodríguez A. (2021). Uniform performance of mammal detection methods under contrasting environmental conditions in Mediterranean landscapes. Ecosphere.

[53] Steinbeiser C.M., Kioko J., Maresi A., Kaitilia R., Kiffner C. (2019). Relative abundance and activity patterns explain method-related differences in mammalian species richness estimates. J. Mammal..

[54] Abrams J.F., Hörig L.A., Brozovic R., Axtner J., Crampton-Platt A., Mohamed A., Wong S.T., Sollmann R., Yu D.W., Wilting A. (2019). Shifting up a gear with iDNA: From mammal detection events to standardised surveys. J. Appl. Ecol..

[55] Espartosa K.D., Pinotti B.T., Pardini R. (2011). Performance of camera trapping and track counts for surveying large mammals in rainforest remnants. Biodivers. Conserv..

[56] Torrents-Ticó M., Rich L., McNutt J.W., Nthomiwa M., Mothala M., Motsamai G., Jordan N.R. (2017). On the right track? Comparing concurrent spoor and camera-trap surveys in Botswana. Afr. J. Wildl. Res..

[57] Pineda E., Lobo J.M. (2012). The performance of range maps and species distribution models representing the geographic variation of species richness at different resolutions. Glob. Ecol. Biogeogr..

[58] Hurlbert A.H., White E.P. (2005). Disparity between range map- and survey-based analyses of species richness: Patterns, processes and implications. Ecol. Lett..

[59] Tingley M.W., Nadeau C.P., Sandor M.E. (2020). Multi-species occupancy models as robust estimators of community richness. Methods Ecol. Evol..

[60] Colyn R.B., Radloff F.G.T., O’Riain M.J. (2018). Camera trapping mammals in the scrubland’s of the Cape Floristic Kingdom—The importance of effort, spacing and trap placement. Biodivers. Conserv..

[61] Gotelli N.J., Chao A., Levin S.A. (2013). Measuring and estimating species richness, species diversity, and biotic similarity from sampling data. Encyclopedia of Biodiversity.

[62] Rockhill A.P., Sollman R., Powell R.A., DePerno C.S. (2016). A comparison of survey techniques for medium- to large-sized mammals in forested wetlands. Southeast. Nat..

[63] Hausser Y., Tagand R., Vimercati E., Mermod S., Fischer C. (2016). Comparing survey methods to assess the conservation value of a community-managed protected area in western Tanzania. Afr. J. Ecol..

[64] Costa A.R.C., Passamani M., da Cunha R.G.T. (2019). Survey of medium-sized and large mammals in semideciduous Atlantic Forest patches near Alfenas, southern Minas Gerais, Brazil. CheckList.

[65] Naing H., Fuller T.K., Sievert P.R., Randhir T.O., Po S.H.T., Maung M., Lynam A.J., Htun S., Thaw W.N., Myint T. (2015). Assessing large mammal and bird richness from camera-trap records in the Hukaung Valley of northern Myanmar. Raffles Bull. Zool..

[66] Mcfadden-Hiller J.E., Hiller T.L. (2015). Non-Invasive survey of forest carnivores in the northern cascades of Oregon, USA. Northwest. Nat..

[67] Weiskopf S.R., McCarthy K.P., Tessler M., Rahman H.A., McCarthy J.L., Hersch R., Faisal M.M., Siddall M.E. (2018). Using terrestrial haematophagous leeches to enhance tropical biodiversity monitoring programmes in Bangladesh. J. Appl. Ecol..

[68] Brocardo C.R., Rodarte R., Bueno R.D.S., Culot L., Galetti M. (2012). Mamíferos não voadores do Parque Estadual Carlos Botelho, Continuum florestal do Paranapiacaba. Biota Neotrop..

[69] Swan M., Di Stefano J., Christie F., Steel E., York A. (2014). Detecting mammals in heterogeneous landscapes: Implications for biodiversity monitoring and management. Biodivers. Conserv..

[70] Fragoso J.M.V., Levi T., Oliveira L.F.B., Luzar J.B., Overman H., Read J.M., Silvius K.M. (2016). Line transect surveys underdetect terrestrial mammals: Implications for the sustainability of subsistence hunting. PLoS ONE.

[71] Qian H., Deng T., Beck J., Sun H., Xiao C., Jin Y., Ma K. (2018). Incomplete species lists derived from global and regional specimen-record databases affect macroecological analyses: A case study on the vascular plants of China. J. Biogeogr..

[72] Troia M.J., McManamay R.A. (2016). Filling in the GAPS: Evaluating completeness and coverage of open-access biodiversity databases in the United States. Ecol. Evol..

[73] Kays R., Arbogast B.S., Baker-Whatton M., Beirne C., Boone H.M., Bowler M., Burneo S.F., Cove M.V., Ding P., Espinosa S. (2020). An empirical evaluation of camera trap study design: How many, how long and when?. Methods Ecol. Evol..

