# Supplementary material for: Estimates of Species Richness and Composition Depend on Detection Method in Assemblages of Terrestrial Mammals
Source: Animals (Basel). 2021 Jan 14;11(1):186. doi: 10.3390/ani11010186 (PMC7830977; doi:10.3390/ani11010186)
Supplement: Supplementary file 1 [file animals-11-00186-s001.pdf]

Supplementary Materials

Estimates of species richness and composition depend on detection method in assemblages of terrestrial mammals

Bruno D. Suárez-Tangil[1] and Alejandro Rodríguez[1,*]

[1]Department of Conservation Biology, Estación Biológica de Doñana – CSIC, Américo Vespucio 26, 41092 Sevilla, Spain

*Correspondence: Alejandro Rodríguez · alrodri@ebd.csic.es

Figure S1. Mean (±SD) mammal species richness of local mammal assemblages obtained from four survey methods and the multi-method survey in each session. A: Year 1, Spring; B: Year 1, Autumn; C: Year 2, Spring; D: Year 2, Autumn. n=24 sampling units for each method.

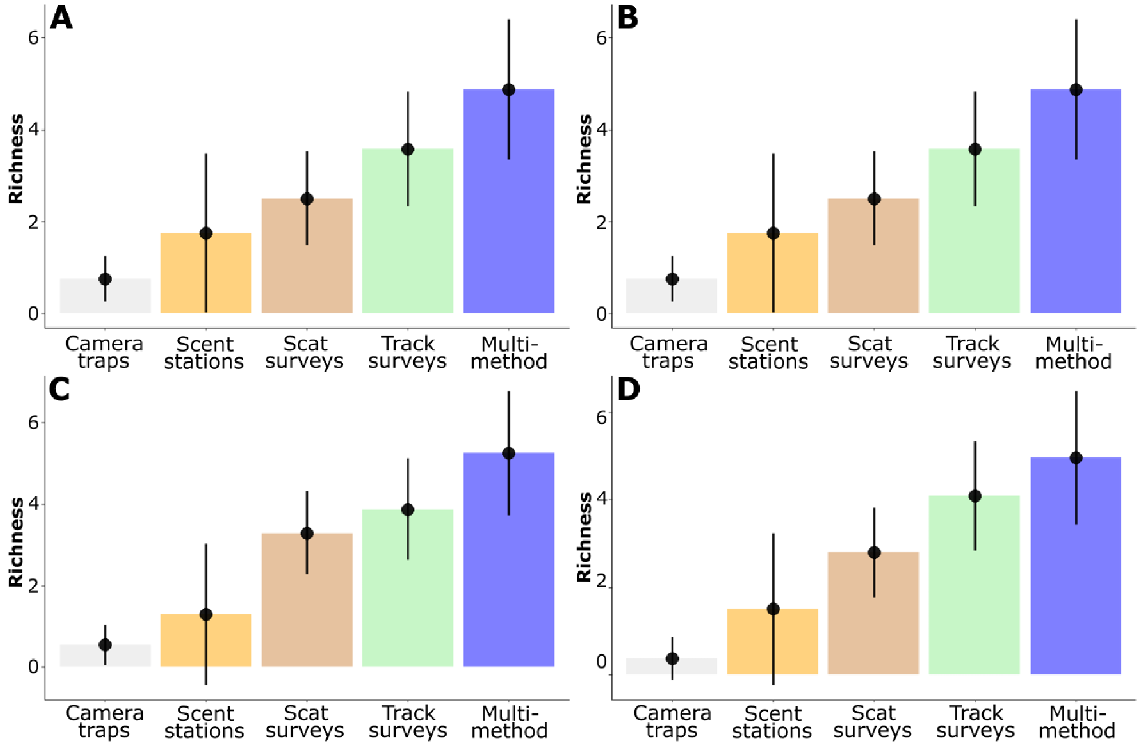

Figure S2. Odds ratio, as an index of the relative efficiency of survey methods for describing species richness. Odds ratio indicates how efficient each method is for detecting all species present in each local assemblage as compared to the reference method, which is assigned to camera traps (odds ratio = 1). Values >1 (dotted line) indicate than the focal method performs better than camera traps.

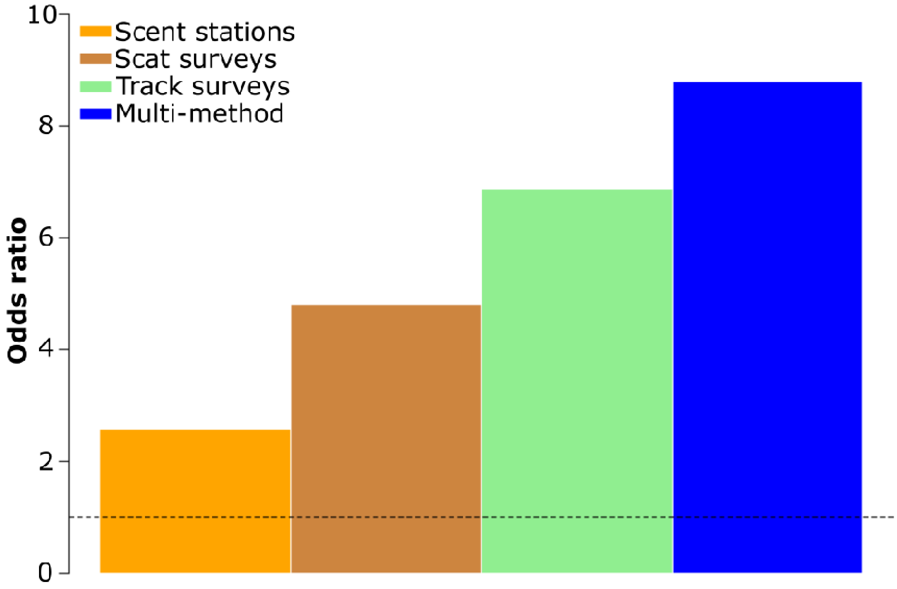

Figure S3. Consistency across sessions of odds ratios quantifying between-method differences in detection of all species present in each local assemblage as compared to the reference method. The reference method was assigned to camera traps (odds ratio = 1). Values >1 (dotted line) indicate that the focal method performs better than camera traps.

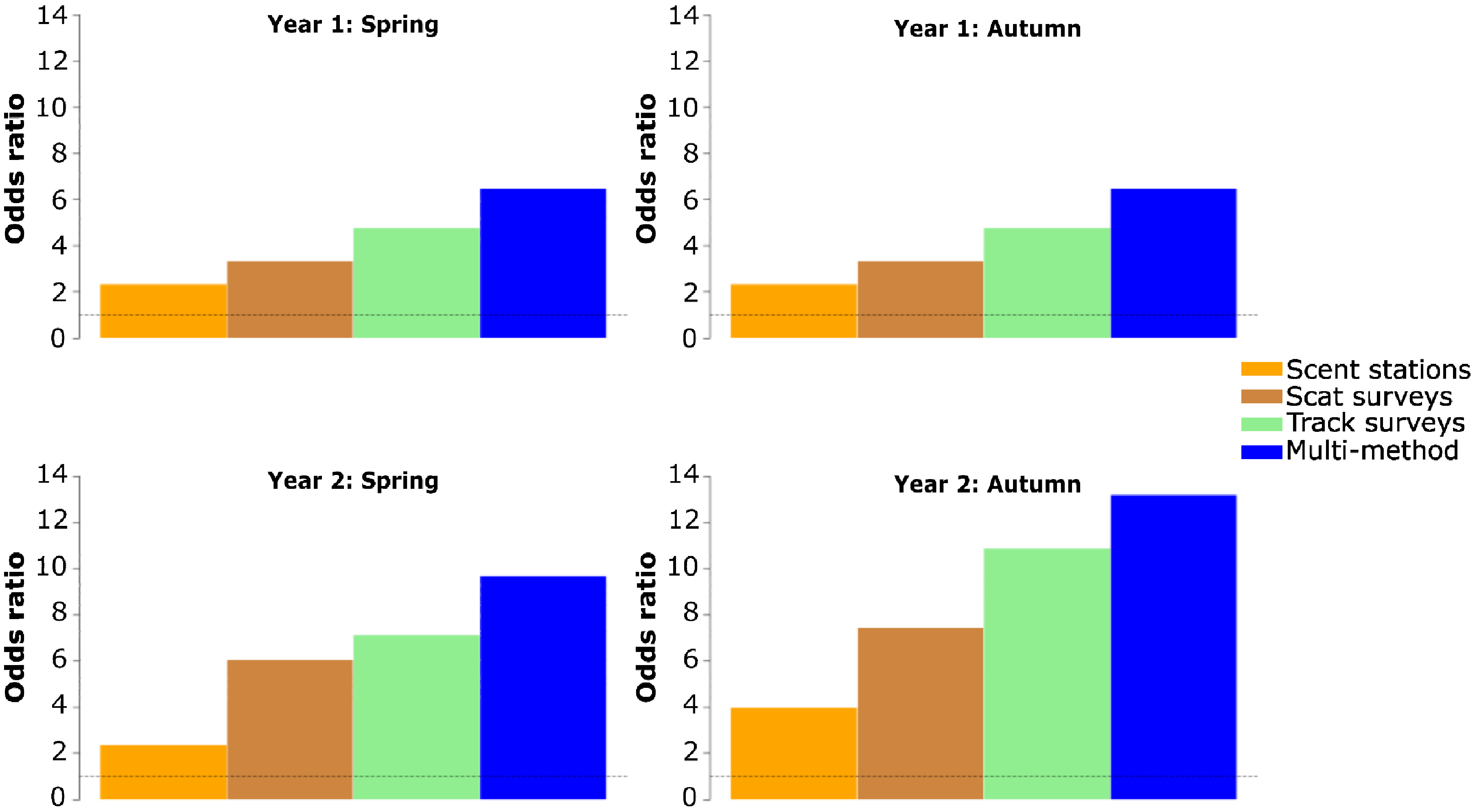

Table S1. Observed *vs.* *e*stimated mammal **s**pecies richness obtained from each survey method. Observed richness is the cumulative number of species over 24 spatial replicates over 4 temporal replicates. From these data, richness was estimated using the iChao2 index [43]. Except for scat surveys, estimated species richness exceeded the regional species pool known to occur in the study area (13 species; [37]).

	**Spatial replicates**		**Temporal replicates**	
Survey method	Observed	Estimated (±SE)	Observed	Estimated (±SE)
Camera traps	9	14.8 ± 5.7	9	13.7 ± 4.5
Scent stations	11	20.6 ± 8.4	11	20.5 ± 10.9
Scat surveys	12	11.0 ± 0.5	12	12.5 ± 2.3
Track surveys	13	15.9 ± 3.7	13	16.4 ± 4.5

Table S2. Pairwise comparisons of the species richness described by different detection methods using Tukey and Kramer test with Tukey distance approximation (α = 0.05).

Pairwise comparison	Statistic	P
Camera traps – Scent stations	5.26	0.002
Camera traps - Scat surveys	12.42	<0.001
Camera traps - Track surveys	17.74	<0.001
Camera traps - Multi-method	21.92	<0.001
Scent stations - Scat surveys	7.17	<0.001
Scent stations – Track surveys	12.48	<0.001
Scent stations - Multi-method	16.67	<0.001
Scat surveys – Track surveys	5.13	0.002
Scat surveys - Multi-method	9.50	<0.001
Track surveys - Multi-method	4.18	0.026

Table S3. Pairwise differences in species richness during the sampling period. Comparisons in bold showed consistent differences in species richness regardless session. *Pairwise comparisons between the survey methods were performed using a Tukey and Kramer test with Tukey distance approximation, employing the Bonferroni correction (α = 0.0125).

	**Consistency**							
	**Year 1: Spring**		**Year 1: Autumn**		**Year 2: Spring**		**Year 2: Autumn**	
Pairwise comparison	Statistic*	P	Statistic*	P	Statistic*	P	Statistic*	P
Camera traps – Scent stations	2.34	0.464	2.93	0.232	2.09	0.576	3.21	0.154
Camera traps-Scat surveys	**5.65**	**0.001**	**5.16**	**0.002**	**7.25**	**<0.001**	**6.63**	**<0.001**
Camera traps – Track surveys	**9.47**	**<0.001**	**8.15**	**<0.001**	**8.17**	**<0.001**	**9.48**	**<0.001**
Camera traps - Multi-method	**10.99**	**<0.001**	**11.12**	**<0.001**	**10.86**	**<0.001**	**10.91**	**<0.001**
Scent stations –Scat surveys	3.31	0.132	2.23	0.514	5.16	0.002	3.42	0.111
Scent stations – Track surveys	**7.14**	**<0.001**	**5.22**	**0.002**	**6.08**	**<0.001**	**6.26**	**<0.001**
Scent stations - Multi-method	**8.65**	**<0.001**	**8.19**	**<0.001**	**8.76**	**<0.001**	**7.70**	**<0.001**
Scat surveys – Track surveys	3.83	0.053	2.99	0.214	0.92	0.967	2.85	0.260
Scat surveys - Multi-method	5.34	0.002	5.96	<0.001	3.60	0.080	4.28	0.021
Track surveys - Multi-method	1.51	0.822	2.98	0.218	2.68	0.318	1.44	0.848

Table S4. Effect of survey method on species richness for each session. (a) Competitive generalised linear models ($\Delta AIC_c \leq 2$) ordered by the fit statistic AIC_c. (b) Parameter estimates of the resulting averaged model. The method 'Camera trap' is included in the intercept. The level 'Sierra Morena' is also included in the intercept for analyses where the factor 'Landscape' was retained in competitive models. AIC_c: corrected Akaike Information Criterion; w_i: Akaike weights.

Year 1: Spring

	Model	df	AIC_c	ΔAIC_c	w_i
(a)	Survey method	5	401.0	0.00	0.705
	Survey method + Landscape	7	402.7	1.74	0.295

(b)	Effect	Coefficient	SE	P
	Intercept	-0.314	0.250	0.215
	Scent station	0.778	0.293	0.009
	Scat survey	1.386	0.271	<0.001
	Track survey	1.903	0.260	<0.001
	Multi-method	2.101	0.257	<0.001
	Guadiamar agroecosystem	-0.207	0.127	0.106
	Doñana	-0.122	0.124	0.328

Year 1: Autumn

	Model	df	AIC_c	ΔAIC_c	w_i
(a)	Survey method	5	385.6	0.00	0.882

(b)	Effect	Coefficient	SE	P
	Intercept	-0.288	0.236	0.222
	Scent station	0.847	0.282	0.003
	Scat survey	1.204	0.269	<0.001
	Track survey	1.564	0.259	<0.001
	Multi-method	1.872	0.253	<0.001

Year 2: Spring

	Model	df	AIC_c	ΔAIC_c	w_i
(a)	Survey method + Landscape	7	391.4	0.00	0.822

(b)	Effect	Coefficient	SE	P
	Intercept	-0.422	0.285	0.139
	Scent station	0.869	0.330	0.009
	Scat survey	1.805	0.299	<0.001
	Track survey	1.968	0.296	<0.001
	Multi-method	2.271	0.291	<0.001
	Guadiamar agroecosystem	-0.332	0.132	0.012
	Doñana	-0.273	0.130	0.035

Year 2: Autumn

	Model	df	AIC_c	ΔAIC_c	w_i
(a)	Survey method	5	382.3	0.00	0.660
	Survey method + Landscape	7	383.6	1.32	0.340

	Effect	Coefficient	SE	P
(b)				
	Intercept	-0.939	0.341	0.006
	Scent station	1.386	0.373	<0.001
	Scat survey	2.008	0.355	<0.001
	Track survey	2.388	0.348	<0.001
	Multi-method	2.582	0.346	<0.001
	Guadiamar agroecosystem	-0.235	0.135	0.085
	Doñana	-0.148	0.132	0.269
